# The Evolving Role of Artificial Intelligence in Medical Genetics: Advancing Healthcare, Research, and Biosafety Management

**DOI:** 10.3390/genes17010006

**Published:** 2025-12-19

**Authors:** Ying-Cheng Wu, Nan Tuo, Guoming Shi, Ka Li, Zhenju Song, Yanying Li

**Affiliations:** 1 Shanghai Medical College, Fudan University, Shanghai 200032, China; 2 Clinical Research Centre, The International Peace Maternity and Child Health Hospital, Shanghai Jiao Tong University School of Medicine, Shanghai 200030, China; 3 Department of Research, Zhongshan Hospital, Fudan University, Shanghai 200032, China

**Keywords:** artificial intelligence, medical genetics, digital twin, pharmacogenomics, biosecurity

## Abstract

The integration of artificial intelligence (AI) with medical genetics is transforming healthcare by addressing the analytical challenges posed by the vast complexity of multi-omics data. This review explores the synergistic convergence of these fields, highlighting AI’s transformative role in enhancing diagnostic precision, enabling non-invasive molecular profiling through imaging-genetics, and advancing predictive and personalized medicine via polygenic risk scores and pharmacogenomics. AI is also emerging as a powerful generative tool in therapeutic design, accelerating drug discovery, protein engineering, and precision gene editing. However, this powerful synergy introduces significant ethical, regulatory, and biosecurity challenges, including data privacy, algorithmic bias, and the dual-use risks of AI-enabled genetic engineering. The future envisions a responsible co-evolution, with multimodal AI and the concept of the Digital Twin driving precision medicine, underpinned by interdisciplinary collaboration to ensure fairness, transparency, and societal trust. This article charts the current landscape and proposes actionable directions, emphasizing the need for robust governance to harness AI’s potential while mitigating its risks for the benefit of human health.

## 1. Introduction

The convergence of medical genetics and artificial intelligence stands as a prime example of an evolution that has been seen in biomedical sciences over the last few decades [1,2]. This convergence of two disciplines was not an accident but a co-evolution that was led by the converging trajectory of data production and processing power [3]. The revolution in genomics from the concept of mendelian genes to that of high-throughput multi-omics had led to a data-dump of unprecedented proportions [4,5,6]. The challenge of data analysis that came along with the growth in data size and complexity could not be met by existing algorithms and hence demanded new technologies [7]. At the same time, the revolution that began from very basic expert systems led to the advent of very complex artificial intelligence through deep-learning algorithms that perfectly meet the above-mentioned demand [8]. This article proposes to track the evolution of these two disciplines and shed light on the combined capabilities of these two disciplines that have led to a revolution in research and implementation that raises burning challenges from an ethics and security perspective that needs to be navigated, thereby outlining the revolution that these two disciplines may bring in maturing an understanding of human disease in future.

The intersection of medical genetics and artificial intelligence represented much more than the crossing of two rapidly progressing fields. Rather, it represented the establishment of a deeply symbiotic relationship. The challenge of data complexity presented by multi-omics in the first area of research became the prime motivator for the other [6]. The challenge posed by the genomics revolution to analysis presented an ideal fit with the capabilities of the artificial intelligence revolution [9].

This synergy has catalyzed a fundamental shift in the scientific method as applied to genetics [10]. The traditional scientific model of hypothesis testing against a given gene or set of genes based on observation has been expanded and in some cases overtaken by a hypothesis-generating model of research [10]. In the new model of research, large data sets submitted to analysis by artificial intelligence algorithms yield new and unexpected correlations and patterns. These data-driven findings then form the basis of new hypothesis testing by human scientists [10]. In this new arena, AI solutions act as sophisticated tools for pattern recognition and optimization, uncovering correlations within data sets that human intelligence might miss. Consequently, the field of medical genetics is evolving from a primarily descriptive science toward one that is increasingly prescriptive and engineered.

### 1.1. Enhancing Diagnostic Yield in Genetic Disease

For patients with rare genetic disorders, the path to a diagnosis is often a long and arduous journey known as the diagnostic odyssey [11]. Whole Exome and Whole Genome Sequencing (WES/WGS) offer hope of putting an end to the diagnostic odyssey by providing a full understanding of the patient’s genetic landscape. These sequencings can pose an avalanche of data that may include thousands or indeed million variants [12]. The definitive problem involves isolating the relevant two variants amidst the ocean of benign genetic variation.

**Figure 1:**
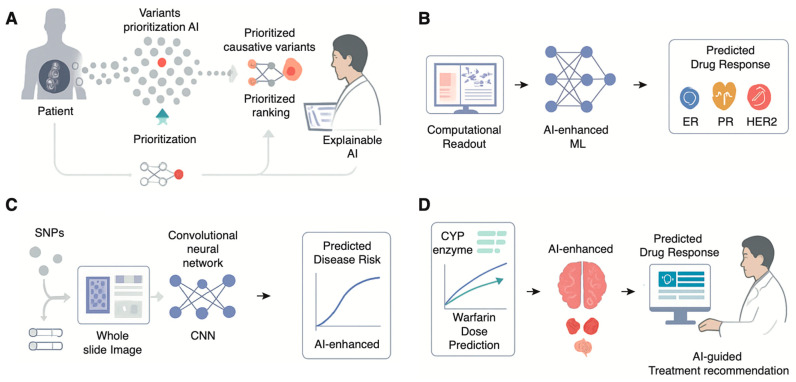
Diverse Applications of Artificial Intelligence in Transforming Medical Genetics. (**A**) Status in breast cancer, thereby guiding treatment decisions. (**B**) Using machine learning to classify drug response across disease subsets. (**C**) Multimodal AI for Predictive Risk Stratification. Deep learning models like Convolutional Neural Networks (CNNs) can integrate both genomic data (e.g., Single Nucleotide Polymorphisms, SNPs) and imaging data (e.g., whole slide images) to generate more accurate, personalized predictions of future disease risk. (**D**) AI-Enhanced Pharmacogenomics (PGx) for Personalized Therapeutics. AI models integrate pharmacogenomic data (e.g., CYP enzyme genotypes affecting warfarin metabolism) to accurately predict an individual’s optimal drug dosage, providing AI-guided treatment recommendations to clinicians to maximize efficacy and minimize adverse reactions.

The effectiveness of these efforts has been made possible only by the intervention of an important new player: artificial intelligence. AI-powered analysis platforms are now increasingly being explored to facilitate diagnosis by automatically ranking candidate genes based on an integration of various lines of evidence [13]. These include the raw genomic data inputted into analysis algorithms, combined with the patient’s phenotype or manifest symptoms and signs, as well as findings from the scientific literature and disease databases, as well as predictive algorithms for assessing the functional significance of a particular gene variant [14]. For instance, a machine-learning algorithm known by the initials AI-MARRVEL or AIM, trained on a huge public database of known cases, can successfully identify the top candidate genes for a given patient with high accuracy in validation cohorts [13]. While currently concentrated in specialized research centers, these tools are gradually moving toward broader clinical utility. These analysis techniques have further demonstrated the capacity to successfully resolve cold cases, patients for whom a diagnosis had long been pending for years, by re-checking genomic data against new disease genes (Figure 1A).

In order to build trust and acceptance within the clinical community, it is important that an emphasis on openness and collaboration be made. Explainable AI (XAI) represents an important part of such an approach [15]. Platforms like Emedgene incorporate XAI to provide a clear, evidence-backed rationale for why a particular variant has been prioritized, allowing clinicians to review the AI’s reasoning and maintain ultimate control over the diagnostic decision [16]. In addition to sequencing solutions on the market based on AI, there have been improvements made in the use of classic genetic testing methods such as chromosomal analysis [17].

### 1.2. The Rise of Imaging-Genetics: Digital Biopsies

One of the most promising uses of artificial intelligence in medicine is the ability to bridge the gap that exists between a patient’s phenotype and genotype by performing “digital biopsies” on routine images of a patient’s medical data [18]. This includes two rapidly emerging fields of medicine: computational pathology and radiogenomics [19] (Table 1).

Computational pathology utilizes the power of AI and more specifically deep learning to analyze digitalized histopathological images of Whole Slide Images. This analysis enables computers to automate tasks that had been carried out by pathologists on a subjective basis until now. These include tumor assessment, tumor grading, determination of the count of mitoses, determination of the expression of certain biomarkers, and others [20]. Furthermore, another area of immense significance that has been covered by computational pathology research based on the power of artificial intelligence relates to the determination of the tumor’s molecular characteristics from a routine H&E stain [21,22,23]. These patterns that contribute towards specific mutations in genes such as TP53, BRAF, PIK3CA can only be detected by computers or artificial intelligence algorithms designed on the principles of deep leaning. These mutations possess immense significance due to the possibility of becoming a rapid and affordable prescreening technique by which pathologists may identify high-priority cases that fit the expense of primary screening by directed sequencing analysis. While computational pathology serves as a rapid prescreening technique for sequencing, radiogenomics extends this same logic of risk reduction and non-invasive molecular inference to the level of whole-body imaging.

Radiogenomics follows a similar concept but incorporates radiological images like Magnetic Resonance Imaging (MRI), Computed Tomography scans, or Positron Emission Tomography scans as input [24]. These images are processed to identify correlations with the data obtained from genes by deep learning algorithms [18]. Radiogenomics enables the non-invasive determination of vital molecular details such as tumor subtypes, primary mutations like Estrogen Receptor Status, Progesterone Receptor Status, and HER2 Status in the case of breast cancer, and even prognoses without requiring an additional biopsy by relying on routine scans [25]. By extracting genetic-level information from routine, lower-cost data sources like pathology slides and medical scans, AI is poised to democratize access to molecular diagnostics, which could be particularly impactful in resource-limited settings where advanced sequencing technologies are not readily available (Figure 1B) [26].

### 1.3. Predictive and Prophylactic Medicine

AI is shifting the focus of medical genetics from diagnosing existing diseases to predicting future risk, enabling a more proactive and preventive approach to healthcare [27]. This transition can particularly be observed within the evolution of Polygenic Risk Scores and within the realm of prenatal analysis. Polygenic Risk Scores are an intricate approach towards the assessment of an individual’s genetic predisposition towards various complex ailments like coronary heart disease, Type 2 Diabetes, and Alzheimer’s disease [28]. The classic model of PRS assessment can be defined as the summation of the joint impacts of thousands or even million-strong single-nucleotide polymorphisms of the genetic makeup of an individual that have been ascertained from Genome-wide association scans [29]. However, these standard PRS models are linear and fail to capture more complex genetic effects, such as interactions between genes (epistasis). Machine learning-enhanced PRSs (ML-PRSs) are emerging as a superior alternative [30]. By using more sophisticated, non-linear algorithms like gradient boosting or neural networks, ML-PRS models can account for these complex interactions and integrate a wider range of predictive features [31]. Studies have consistently shown that ML-PRS models outperform standard PRS in predicting disease risk and related biomarkers, offering a more accurate tool for patient stratification and guiding preventive strategies (Figure 1C) [32].

### 1.4. Tailoring Therapeutics with Pharmacogenomics (PGx)

The ultimate goal of personalized medicine is to deliver the right drug, at the right dose, to the right patient. Pharmacogenomics is an area of research that deals with understanding an individual’s genetic makeup and relating it to his/her drug response. The area of Pharmacogenomics has recently adopted artificial intelligence. The capabilities of artificial intelligence and machine learning algorithms include understanding the huge amount of data that includes an individual’s genetic makeup as well as his/her drug response outcomes. The algorithms identify high-risk patients who may suffer from adverse drug reactions [33]. These models’ prognostic capabilities can be improved by unifying multi-omics information. Using a patient’s gene expression data, genome information, and protein information altogether via more advanced artificial intelligence models provides a much better biological perspective than those models that simply analyze the patient’s genes. For example, it has been observed that combining gene expression data with gene mutation data improved the effectiveness of warfarin dosing predictions [34,35]. Drug-target interactions at the molecular level can be represented by artificial intelligence models themselves. These could inform why a given patient’s treatment may have been ineffectual against them due to a particular mutation within the target protein that exists within the tumor. These models offer predictive capabilities that are enabling physicians to personalize treatment decisions for higher effectiveness and reduced adverse outcomes (Figure 1D).

## 2. Engineering Biology: AI in Therapeutic Design and Genetic Modification

**Figure 2:**
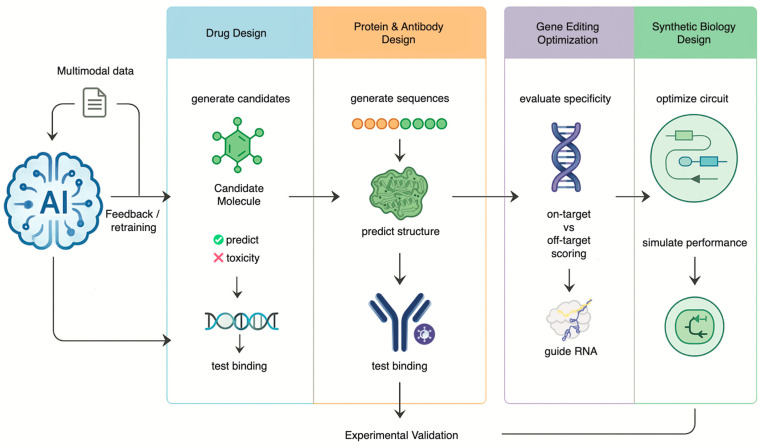
AI as a Generative Engine for Engineering Biology This diagram shows AI driving an iterative design-build-test-learn cycle. Trained on multimodal data, the AI generates and optimizes novel drug molecules, proteins, guide RNAs, and synthetic biological circuits. Results from experimental validation are fed back to continuously retrain and improve the model’s performance.

Beyond its role as a powerful analytical engine, AI is undergoing a profound transformation into a generative engine in biomedical science [36]. This generative leap is moving biology from a science of observation and discovery to a true engineering discipline [36]. AI is no longer just predicting outcomes based on existing data; it is learning the fundamental rules of biological systems and using that knowledge to design novel molecules, proteins and genetic systems based on patterns derived from vast training datasets [37]. This shift is most evident in the realms of drug discovery, protein engineering, gene editing and synthetic biology, where AI is enabling the creation of therapeutic solutions that may be superior to those found in nature (Figure 2).

### 2.1. Accelerating Drug Discovery and Development

The conventional drug discovery pipeline is known to be very expensive and full of failures. The usual procedure involves screening compounds in order to obtain promising “hits.” The procedure may take over a decade and cost billions of dollars. The success rate in the usual drug discovery pipeline is very low. The role of AI in drug discovery may revolutionize the whole process [38].

Today, artificial intelligence solutions have been trained on massive multivariate data sets—including omics data, scientific literature, clinical trial data, and compound libraries—to discover new drug targets, model molecular physicochemical properties such as bioactivity and toxicity, and design new synthesis routes for new compounds. This marks a shift from screening to intelligent design. The effects of such advances are now being seen in the industry. The successful utilization of these platforms by companies such as Insilico Medicine and Exscientia has led to the discovery of new drug therapies designed from scratch that have successfully been put through clinical trials on human subjects much quicker and at a lower cost than before. In addition, drug repositioning using AI has been found to be particularly successful as seen by the swift selection of Baricitinib, an Inhibitor of Janus Kinase (JAK), as a treatment for COVID-19 (Figure 2) [38,39].

### 2.2. Generative AI in DNA, Protein and Antibody Engineering

Advanced displays of AI’s generative capacity relates to de novo protein and antibody design. The use of generative models of AI of a kind similar to those that power chatbots that use Large Language Models (LLM) is the learning of the language or grammar of protein/DNA biology—a set of very complex rules which dictate how a linear array of amino acids translates into a real three-dimensional structure, including the algorithms covering alphafold3 and evo [36,40,41].

Through knowledge of these rules, it is now possible to create new amino acid structures for proteins that have never appeared in nature but have been modeled to adopt stable conformations and undertake specific biological functions. The potential here is enormous. For example, scientists were able to create new generations of antimicrobial peptides using generative models of artificial intelligence that can tackle multidrug-resistant bacteria. In another revolutionary area of research [37], AI models were used to design peptides that can bind successfully to and degrade “undruggable” proteins with unstable conformations or undefined binding pockets that had long been resistant to drug design. The idea appears promising for proteins that may be involved in various forms of cancer and neurodegenerative diseases such as Huntington’s disease [42]. Although antibody–antigen predictions remain particularly challenging, with current methods exhibiting failure rates exceeding 50%, interestingly, AlphaFold 3 consistently demonstrates superior accuracy across the majority of tasks, compared with all other models [43].

This transformative model of design is also being applied to the design of therapeutic antibodies. In contrast to the painstaking effort of screening against very large libraries of candidate antibodies, “zero-shot”, de novo design of antibodies against a particular antigen target has now become practical using generative models of AI [36]. The model uses a very strong positive feedback loop that cycles seamlessly from computational design work in silico to high-throughput experimental testing in the laboratory. The model produces thousands of candidate antibody designs that can be produced very rapidly in the laboratory. The data from these lab tests are used to inform and improve the next cycle of designs as the model learns from both positive and negative outcomes. This strong collaboration has already led to the design and production of antibodies that bind targets with binding affinities that exceed the affinities of drugs such as trastuzumab, already on the market as therapeutic antibodies [36].

### 2.3. Refining Gene Editing with Precision

The development of CRISPR-based gene editing has opened up extraordinary possibilities for treating genetic diseases [44]. However, the safety and efficacy of these therapies depend critically on the precise design of the tools used, particularly the guide RNA (gRNA) that directs the Cas enzyme to the correct location in the genome. Designing an optimal gRNA involves maximizing its on-target editing efficiency while minimizing the risk of it cutting at unintended, similar-looking sites elsewhere in the genome (off-target effects).

AI has become an integral part of solving such a complex optimization problem [45]. The data from these large-scale experiments have been used to train deep learning algorithms that accurately predict gRNA on-target activity as well as co-designing RNA and genome editing complex [46]. These algorithms have the capacity to analyze not only the gRNAs used but also the surrounding environment within the chromatin that determines accessibility to the Cas enzyme. At the same time, these algorithms have become vital for an extensive analysis of the whole genome to identify off-target sites of high specificity gRNAs that have been chosen by scientists.

The role of AI not only involves enhancing existing solutions but also working towards creating new ones. For instance, AI has been applied in the design of novel base editors with improved efficiency through protein engineering [47]. In an impressive demonstration of capabilities of AI in creation, an LLM-assisted approach enabled the design of OpenCRISPR-1, the first working gene editor that has been designed by AI from scratch as a novel solution [45]. OpenCRISPR-1 was found to possess on-target editing efficiencies comparable to that of the commonly used naturally occurring SpCas9 enzyme but with improved specificity and reduced off-targeting in human cells.

## 3. Navigating the New Frontier: Biosafety, Governance, and Ethical Imperatives

The transformative power of AI within medical genetics comes with immense challenges from an ethical, legal, security perspective. This “pacing problem,” where the pace of technological change continues to rapidly outpace the capacity of effective regulation and oversight, has become an area of acute concern [48,49,50]. Successfully navigating this new frontier requires a proactive, socio-technical approach that embeds principles of safety, fairness, and accountability into the fabric of research and development. A failure to address these issues risks creating a trust deficit with the public, which could undermine the entire enterprise by hindering data sharing and impeding the equitable deployment of these powerful technologies [50].

### 3.1. Data Privacy and Security in the AI Era

Genomic data is unique among health information due to its inherent and permanent identifiability. With the exception of identical twins, an individual’s DNA sequence is a unique identifier that also contains sensitive information about their health risks, ancestry, and biological relatives [50]. Consequently, traditional data anonymization techniques, such as removing names and addresses, are often insufficient to guarantee privacy [50]. This poses a paradoxical challenge towards fulfilling the scientific imperative for data sharing in accelerating scientific research initiatives. In addressing this impasse for data privacy preservation and within the scientific community’s imperative for data sharing in accelerating research initiatives, various measures are being developed. These measures include controlled-access databases secured by data ‘enclaves,’ whereby scientists apply for data analysis from de-identified data sources via a controlled scientific analysis environment. But more radical interventions may be derived from privacy-enhancing technologies.

Federated Learning (FL) is a particularly promising privacy-enhancing technologies for genomic research [51,52]. FL can thus be considered a decentralized form of machine learning in which, instead of transferring the precious data to the model, the model itself is transferred to the data. In a normal FL setup, various organizations such as hospitals or research organizations work in unison to develop a global model of an artificial intelligence system without transferring their raw data to each other. The system only transfers the model’s updates in terms of gradients or model weights to a central hub for processing. In turn, various organizations work for many cycles to develop an optimized global model that relies on all the decentralized data without transferring the precious data. Although FL is not a foolproof system as it can still be breached by an intelligent inference attack, it marks a milestone change towards privacy by design and usually works in conjunction with various privacy-enhancing technologies such as differential privacy and secure two-party computation.

### 3.2. Algorithmic Bias and Health Equity

One of the most important moral dilemmas that artificial intelligence in the healthcare industry encounters is that of bias. The primary factor that determines the effectiveness of an artificial intelligence system is the data on which it has been trained. If it fails to represent the population on which it is being applied, it may result in inequity as well as negative consequences [53].

In the realm of genomics, it is an especially pressing issue. In the largest genomic databases and those of genome-wide association studies (GWASs), the majority of information primarily pertains to those of European ancestry. A balanced discussion of these challenges is essential for credibility. For example, AI naïve tools such as DeepVariant demonstrated robust performance by maintaining superior callset quality across a range of factors such as sequence coverage (15–50×) and sequencing method wide coverage [54]. As such, an AI system trained on such imbalanced data inevitably yields poorer outcomes for those of unrepresented ancestral groups—such as those of African, Asian, or Indigenous backgrounds [52]. This can lead to a dangerous cycle where AI not only reflects but actively amplifies existing health disparities. For example, a polygenic risk score for a specific disease developed using European data may systematically underestimate risk in an African population, leading to missed opportunities for preventive care and worse health outcomes.

Mitigating this bias requires a concerted, multi-pronged effort. To mitigate this, researchers are exploring concrete technical strategies such as reweighting methods during model training and adversarial debiasing to minimize performance disparities. However, addressing the deeper structural factors impeding diverse data collection remains paramount [53]. The most crucial step is to increase the diversity of genomic datasets through targeted recruitment and inclusive research practices. From a technical perspective, researchers are developing fairness-aware algorithms that are designed to minimize performance disparities across different demographic groups. Furthermore, rigorous auditing of AI models for bias before and after deployment must become standard practice. Ultimately, ensuring that the benefits of AI-enabled genetics are accessible to all requires a commitment to health equity at every stage of the technology’s lifecycle, from data collection to clinical implementation.

### 3.3. The Dual-Use Dilemma: Biosecurity in an Age of AI-Enabled Genetic Engineering

The same set of powerful artificial intelligence technologies that are accelerating the design of life-saving medications also pose the potential for malicious use. This dual-use problem, wherein technologies designed for positive uses may be applied for negative purposes, raises important biosecurity concerns. Specific examples of potential misuse include the AI-assisted design of pathogens with enhanced virulence or the generation of sequences that evade current screening protocols [55,56,57]. The integration of AI and biotechnology could lower the technical barrier for state or non-state actors to develop sophisticated biological weapons [48,49].

These may include the production of proteins with increased toxicity by using artificial intelligence algorithms. Another area may include the design of new microorganisms with higher virulence and transmissibility. Finally, another area may include the design of new microorganisms that can evade existing immunities as well as treatments. Large language models may have the potential of providing an adversary with a set of instructions that may include launching a biological attack. Additionally, artificial intelligence algorithms may include designing harmful DNA strands that may still evade the screening process followed by existing commercial DNA synthesis companies [49].

Addressing these AI-enabled biorisks requires a multi-layered defense strategy. This includes technical, policy, and community-based safeguards. Technical safeguards involve building guardrails directly into AI models, such as refusal mechanisms that prevent them from responding to dangerous queries, and developing more advanced, AI-powered screening tools to detect novel sequences of concern. Policy measures include strengthening national and international oversight of DNA synthesis providers and establishing clear guidelines for responsible AI development in the life sciences. Fostering a culture of responsibility and security awareness within the scientific community is also critical to ensure that researchers are cognizant of the dual-use potential of their work and take steps to mitigate risks [49].

## 4. Challenges and Future Directions

**Figure 3:**
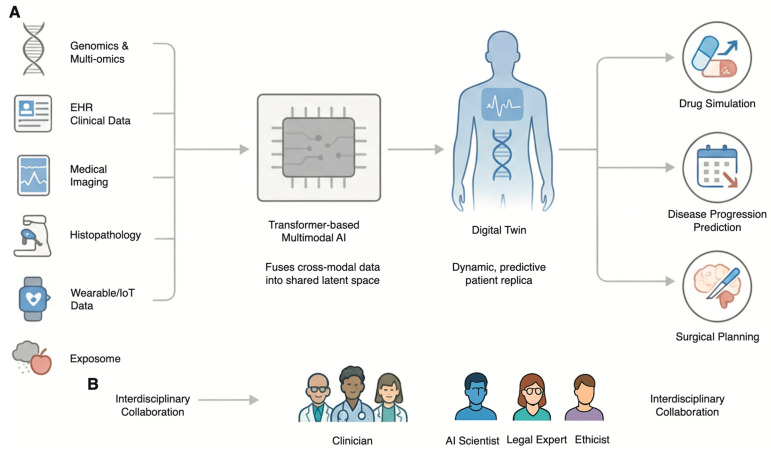
The Future of AI in Genetics: The Digital Twin and Collaborative Framework. (**A**) The Digital Twin as the Future of Precision Medicine. Multimodal data—including Genomics and Multi-omics, EHR Clinical Data, Medical Imaging, Histopathology, Wearable/IoT Data, and the Exposome—is integrated by a Transformer-based Multimodal AI. This creates a dynamic, predictive patient replica known as the Digital Twin. This virtual model is used to simulate treatments (Drug Simulation), forecast future health events (Disease Progression Prediction), and guide medical procedures (Surgical Planning). (**B**) The Imperative of Interdisciplinary Collaboration. The safe and effective development of the Digital Twin and other advanced AI applications requires deep collaboration among Clinicians, AI Scientists, Legal Experts, and Ethicists throughout the entire technology lifecycle.

As artificial intelligence becomes more deeply interwoven with the fabric of medical genetics, the future trajectory points toward a paradigm of deeply integrated, personalized, and predictive healthcare. This evolution is not merely about developing more powerful algorithms but about creating a synergistic ecosystem where technology augments human expertise within a robust framework of responsible innovation. To realize the potential of AI in genetics, a clear roadmap is required. Short-term goals should focus on the rigorous validation of AI tools in diverse clinical cohorts, while long-term goals must aim for the seamless integration of these tools into electronic health records and clinical workflows (Figure 3A) [58,59,60].

### 4.1. The Emergence of the Digital Twin in Precision Medicine

The eventual integration of AI in the healthcare industry has been termed the Digital Twin or Digital Human Twin (DHT) concept. The digital twin incorporates much more than an electronic healthcare record. While currently an aspirational concept facing technical barriers, it holds the potential to simulate therapeutic interventions in silico. In fact, it represents an accurate virtual replica of an individual that changes continually in real-time. The model functions as the overall unifying system for precise healthcare that incorporates every disparate application of artificial intelligence [61,62].

The digital twin model will be created from an intricate mesh of data from various sources to create an integrated model of the patient. These sources include the primary model based on genomic and multiomics analysis that forms the foundation of the model. This also includes data from the electronic health records (EHRs), as well as data from medical images and real-time physiological data from wearables and the Internet of Things. The model also includes data on the exposome that determines the interactions of genes within the environment [61,62,63].

The power of the digital twin lies in its predictive and simulative capabilities. Clinicians will be able to use this virtual patient to model disease progression, predict the onset of future illnesses years in advance, and, most powerfully, simulate the effects of various therapeutic interventions in silico [61,62]. This allows for the optimization of treatment plans—selecting the most effective drug and dose for an individual’s unique biological makeup—before a single medication is administered, thereby maximizing efficacy and minimizing the risk of adverse effects [61]. This technology is already being pioneered in fields like cardiology, where genotype-specific digital twins of patients’ hearts are used to predict arrhythmia risk and guide surgical planning [64].

### 4.2. The Power of Multimodal AI

The fulfillment of the digital twin vision relies primarily on advances in multimodal AI. These refer to complex AI models that can work on multiple data sources such as genomes, images from the Radiological images repository, slides from the Histopathologic image repository, and text from the electronic health record repository [63,65].

This approach is powerful because it mirrors the cognitive process of a human physician, who naturally synthesizes information from a patient’s history, physical exam, lab results, and imaging studies to arrive at a diagnosis. By learning the complex, cross-modal relationships within this data, multimodal AI models consistently demonstrate superior performance compared to unimodal models that analyze only a single data type [63,65]. For example, a model that combines a patient’s tumor genomics with its appearance on a pathology slide can make more accurate prognostic predictions than a model using either data type alone [66]. The development of foundation models and transformer-based architectures capable of creating a shared latent space for these diverse modalities is a key area of ongoing research that will underpin the future of AI-driven diagnostics [65].

### 4.3. A Call for Interdisciplinary Collaboration

The successful and responsible implementation of AI in medical genetics cannot be an isolated challenge to be met by either computer scientists and/or geneticists. This collaboration is not merely desirable but foundational. No small amount of knowledge of a host of fields of research can be assumed by either these scientists or these geneticists.

The establishment of AI solutions that are technically strong, clinically relevant, ethically sound, and legally valid requires the assembly of a team of experts who represent a “union of interests.” Data scientists and AI developers need to be closely integrated with clinical geneticists and genetic counselors to ensure that the solutions being developed meet clinically relevant needs and can be incorporated without disrupting the existing work flow. Ethicists, lawyers, and social scientists need to be integrated from the very beginning to navigate the complex web of issues of privacy, bias, and accountability. But perhaps most important of all is the engagement of patients and the wider community as partners in the process of developing these technologies in a manner that resonates with societal values and nurtures public trust. In such a manner, by promoting a “common language,” it appears that only a multidisciplinary approach can ensure that the evolution of both AI and genetics occurs in a manner that is both innovative as well as responsible (Figure 3B).

### 4.4. Summary

The convergence of artificial intelligence and medical genetics is catalyzing a revolution in healthcare, with the potential to unlock new scientific discoveries, accelerate the development of novel therapeutics, and deliver a new era of personalized medicine. From deciphering the complexities of the genome to designing life-saving drugs and engineering precise gene therapies, AI is augmenting human capabilities at every turn [58,59].

However, this immense potential is balanced by significant peril. The journey forward must be navigated with a clear-eyed understanding of the risks related to data privacy, algorithmic bias, and biosecurity. The relationship between genetics and AI should not be viewed solely through a technological lens; the unique challenges posed by genomic data are actively pushing the boundaries of AI research, driving innovation in areas like explainability, privacy-preserving methods, and specialized model architectures. This reciprocal influence underscores a truly symbiotic co-evolution.

The ultimate goal should not be a system in which the role of the human expert is displaced by the capabilities of artificial intelligence. Rather, it should be a system in which artificial intelligence serves as an invaluable resource that solves the complexity problem that comes with biological data. This system should also be paired with that knowledge from human experts that comes from the perspective of the patient.

In order to ensure that such an alliance succeeds and benefits the whole of humanity as a whole, it is important that it follows a future that emphasizes a commitment to responsible innovation. The FUTURE-AI principles (Fairness, Universality, Traceability, Usability, Robustness, and Explainability) should serve as essential foundations, rather than optional guidelines, to ensuring this revolution is safe, equitable, and beneficial [67]. By embedding these principles into the entire lifecycle of AI development—from design and validation to regulation and deployment—we can work to ensure that the ongoing revolution in medical genetics is safe, equitable, and profoundly beneficial for generations to come.

## Figures and Tables

**Table 1:** A comparative framework summarizing the key AI technologies used in different applications.

Application Area	Key AI Technologies	Pros/Benefits	Cons/Limitations
Diagnostics	Deep Learning, NLP (e.g., AI-MARRVEL)	Integrates multi-modal data; high accuracy in ranking variants.	Requires large training datasets; interpretability issues; validation needed in diverse populations.
Imaging-Genetics	CNNs (Radiomics, Comp. Pathology)	Non-invasive digital biopsies; lower cost than sequencing; spatial heterogeneity analysis.	Sensitivity/specificity varies; standardization of imaging protocols required; indirect inference of genotype.
Predictive Medicine	Machine Learning (ML-PRS)	Captures non-linear genetic interactions; improved risk stratification for complex diseases.	Major bias toward European ancestry in training data; risk of over-medicalization.
Therapeutics	Generative AI, LLMs	Accelerates drug discovery; de novo protein design; optimizes gene editing specificity.	High computational cost; hallucination of non-functional molecules; dual-use biosecurity risks.

## Data Availability

No new data were created or analyzed in this study. Data sharing is not applicable to this article.

## Abbreviations

AI: Artificial Intelligence; AIM: AI-MARRVEL; AI-MARRVEL: Artificial Intelligence-MARRVEL; CNNs: Convolutional Neural Networks; CT: Computed Tomography; CYP: Cytochrome P450; DHT: Digital Human Twin; EHRs: Electronic Health Records; ER: Estrogen Receptor; FL: Federated Learning; FUTURE-AI: Fairness, Universality, Traceability, Usability, Robustness, and Explainability; gRNA: guide RNA; GWAS: Genome-wide association studies; H&E: Hematoxylin and Eosin; HER2: Human Epidermal growth factor Receptor 2; JAK: Janus Kinase; LLM: Large Language Models; ML-PRS: Machine learning-enhanced Polygenic Risk Scores; MRI: Magnetic Resonance Imaging; PGx: Pharmacogenomics; PR: Progesterone Receptor; PRS: Polygenic Risk Scores; SNP: Single Nucleotide Polymorphisms; WES: Whole Exome Sequencing; WGS: Whole Genome Sequencing; XAI: Explainable AI.

## References

[1] Topol E.J. (2019). High-performance medicine: The convergence of human and artificial intelligence. Nat. Med..

[2] Eraslan G., Avsec Ž., Gagneur J., Theis F.J. (2019). Deep learning: New computational modelling techniques for genomics. Nat. Rev. Genet..

[3] Goodwin S., McPherson J.D., McCombie W.R. (2016). Coming of age: Ten years of next-generation sequencing technologies. Nat. Rev. Genet..

[4] Lander E.S., Linton L.M., Birren B., Nusbaum C., Zody M.C., Baldwin J., Devon K., Dewar K., Doyle M., FitzHugh W. (2001). Initial sequencing and analysis of the human genome. Nature.

[5] Auton A., Brooks L.D., Durbin R.M., Garrison E.P., Kang H.M., Korbel J.O., Marchini J.L., McCarthy S., McVean G.A., Abecasis G.R. (2015). A global reference for human genetic variation. Nature.

[6] Hasin Y., Seldin M., Lusis A. (2017). Multi-omics approaches to disease. Genome Biol..

[7] Karczewski K.J., Snyder M.P. (2018). Integrative omics for health and disease. Nat. Rev. Genet..

[8] LeCun Y., Bengio Y., Hinton G. (2015). Deep learning. Nature.

[9] Zhou J., Troyanskaya O.G. (2015). Predicting effects of noncoding variants with deep learning-based sequence model. Nat. Methods.

[10] Beam A.L., Kohane I.S. (2018). Big Data and Machine Learning in Health Care. JAMA.

[11] Wu A.C., McMahon P., Lu C. (2020). Ending the Diagnostic Odyssey-Is Whole-Genome Sequencing the Answer?. JAMA Pediatr..

[12] Ng P.C., Levy S., Huang J., Stockwell T.B., Walenz B.P., Li K., Axelrod N., Busam D.A., Strausberg R.L., Venter J.C. (2008). Genetic variation in an individual human exome. PLoS Genet..

[13] Mao D., Liu C., Wang L., Ai-Ouran R., Deisseroth C., Pasupuleti S., Kim S.Y., Li L., Rosenfeld J.A., Meng L. (2024). AI-MARRVEL—A Knowledge-Driven AI System for Diagnosing Mendelian Disorders. NEJM AI.

[14] Smedley D., Jacobsen J.O., Jäger M., Köhler S., Holtgrewe M., Schubach M., Siragusa E., Zemojtel T., Buske O.J., Washington N.L. (2015). Next-generation diagnostics and disease-gene discovery with the Exomiser. Nat. Protoc..

[15] Kim H.H., Kim D.W., Woo J., Lee K. (2024). Explicable prioritization of genetic variants by integration of rule-based and machine learning algorithms for diagnosis of rare Mendelian disorders. Hum. Genom..

[16] Meng L., Attali R., Talmy T., Regev Y., Mizrahi N., Smirin-Yosef P., Vossaert L., Taborda C., Santana M., Machol I. (2023). Evaluation of an automated genome interpretation model for rare disease routinely used in a clinical genetic laboratory. Genet. Med..

[17] Yang C., Li T., Dong Q., Zhao Y. (2023). Chromosome classification via deep learning and its application to patients with structural abnormalities of chromosomes. Med. Eng. Phys..

[18] Lambin P., Leijenaar R.T.H., Deist T.M., Peerlings J., de Jong E.E.C., van Timmeren J., Sanduleanu S., Larue R., Even A.J.G., Jochems A. (2017). Radiomics: The bridge between medical imaging and personalized medicine. Nat. Rev. Clin. Oncol..

[19] Wang S., Yang D.M., Rong R., Zhan X., Xiao G. (2019). Pathology Image Analysis Using Segmentation Deep Learning Algorithms. Am. J. Pathol..

[20] Campanella G., Hanna M.G., Geneslaw L., Miraflor A., Werneck Krauss Silva V., Busam K.J., Brogi E., Reuter V.E., Klimstra D.S., Fuchs T.J. (2019). Clinical-grade computational pathology using weakly supervised deep learning on whole slide images. Nat. Med..

[21] Coudray N., Ocampo P.S., Sakellaropoulos T., Narula N., Snuderl M., Fenyö D., Moreira A.L., Razavian N., Tsirigos A. (2018). Classification and mutation prediction from non-small cell lung cancer histopathology images using deep learning. Nat. Med..

[22] Niehues J.M., Quirke P., West N.P., Grabsch H.I., van Treeck M., Schirris Y., Veldhuizen G.P., Hutchins G.G.A., Richman S.D., Foersch S. (2023). Generalizable biomarker prediction from cancer pathology slides with self-supervised deep learning: A retrospective multi-centric study. Cell Rep. Med..

[23] Jang H.J., Lee A., Kang J., Song I.H., Lee S.H. (2020). Prediction of clinically actionable genetic alterations from colorectal cancer histopathology images using deep learning. World J. Gastroenterol..

[24] Guiot J., Vaidyanathan A., Deprez L., Zerka F., Danthine D., Frix A.N., Lambin P., Bottari F., Tsoutzidis N., Miraglio B. (2022). A review in radiomics: Making personalized medicine a reality via routine imaging. Med. Res. Rev..

[25] Liu Q., Hu P. (2023). Radiogenomic association of deep MR imaging features with genomic profiles and clinical characteristics in breast cancer. Biomark. Res..

[26] Kather J.N., Pearson A.T., Halama N., Jäger D., Krause J., Loosen S.H., Marx A., Boor P., Tacke F., Neumann U.P. (2019). Deep learning can predict microsatellite instability directly from histology in gastrointestinal cancer. Nat. Med..

[27] Torkamani A., Wineinger N.E., Topol E.J. (2018). The personal and clinical utility of polygenic risk scores. Nat. Rev. Genet..

[28] Khera A.V., Chaffin M., Aragam K.G., Haas M.E., Roselli C., Choi S.H., Natarajan P., Lander E.S., Lubitz S.A., Ellinor P.T. (2018). Genome-wide polygenic scores for common diseases identify individuals with risk equivalent to monogenic mutations. Nat. Genet..

[29] Choi S.W., Mak T.S., O’Reilly P.F. (2020). Tutorial: A guide to performing polygenic risk score analyses. Nat. Protoc..

[30] Sigurdsson A.I., Louloudis I., Banasik K., Westergaard D., Winther O., Lund O., Ostrowski S.R., Erikstrup C., Pedersen O.B.V., Nyegaard M. (2023). Deep integrative models for large-scale human genomics. Nucleic Acids Res..

[31] Klau J.H., Maj C., Klinkhammer H., Krawitz P.M., Mayr A., Hillmer A.M., Schumacher J., Heider D. (2023). AI-based multi-PRS models outperform classical single-PRS models. Front. Genet..

[32] Gunter N.B., Gebre R.K., Graff-Radford J., Heckman M.G., Jack C.R., Lowe V.J., Knopman D.S., Petersen R.C., Ross O.A., Vemuri P. (2024). Machine Learning Models of Polygenic Risk for Enhanced Prediction of Alzheimer Disease Endophenotypes. Neurol. Genet..

[33] Dsouza V.S., Leyens L., Kurian J.R., Brand A., Brand H. (2025). Artificial intelligence (AI) in pharmacovigilance: A systematic review on predicting adverse drug reactions (ADR) in hospitalized patients. Res. Soc. Adm. Pharm..

[34] Gottlieb A., Daneshjou R., DeGorter M., Bourgeois S., Svensson P.J., Wadelius M., Deloukas P., Montgomery S.B., Altman R.B. (2017). Cohort-specific imputation of gene expression improves prediction of warfarin dose for African Americans. Genome Med..

[35] Zack M., Stupichev D.N., Moore A.J., Slobodchikov I.D., Sokolov D.G., Trifonov I.F., Gobbs A. (2025). Artificial Intelligence and Multi-Omics in Pharmacogenomics: A New Era of Precision Medicine. Mayo Clin. Proc. Digit. Health.

[36] Vecchietti L.F., Wijaya B.N., Armanuly A., Hangeldiyev B., Jung H., Lee S., Cha M., Kim H.M. (2025). Artificial intelligence-driven computational methods for antibody design and optimization. mAbs.

[37] Torres M.D.T., Zeng Y., Wan F., Maus N., Gardner J., de la Fuente-Nunez C. (2024). A generative artificial intelligence approach for antibiotic optimization. bioRxiv.

[38] Niazi S.K. (2025). Artificial Intelligence in Small-Molecule Drug Discovery: A Critical Review of Methods, Applications, and Real-World Outcomes. Pharmaceuticals.

[39] Richardson P., Griffin I., Tucker C., Smith D., Oechsle O., Phelan A., Rawling M., Savory E., Stebbing J. (2020). Baricitinib as potential treatment for 2019-nCoV acute respiratory disease. Lancet.

[40] Abramson J., Adler J., Dunger J., Evans R., Green T., Pritzel A., Ronneberger O., Willmore L., Ballard A.J., Bambrick J. (2024). Accurate structure prediction of biomolecular interactions with AlphaFold 3. Nature.

[41] Nguyen E., Poli M., Durrant M.G., Kang B., Katrekar D., Li D.B., Bartie L.J., Thomas A.W., King S.H., Brixi G. (2024). Sequence modeling and design from molecular to genome scale with Evo. Science.

[42] Chen T., Dumas M., Watson R., Vincoff S., Peng C., Zhao L., Hong L., Pertsemlidis S., Shaepers-Cheu M., Wang T.Z. (2024). PepMLM: Target Sequence-Conditioned Generation of Therapeutic Peptide Binders via Span Masked Language Modeling. arXiv.

[43] Xu S., Feng Q., Qiao L., Wu H., Shen T., Cheng Y., Zheng S., Sun S. (2025). Benchmarking all-atom biomolecular structure prediction with FoldBench. Nat. Commun..

[44] Lee M. (2023). Deep learning in CRISPR-Cas systems: A review of recent studies. Front. Bioeng. Biotechnol..

[45] Ruffolo J.A., Nayfach S., Gallagher J., Bhatnagar A., Beazer J., Hussain R., Russ J., Yip J., Hill E., Pacesa M. (2025). Design of highly functional genome editors by modelling CRISPR-Cas sequences. Nature.

[46] Zhang Z., Jin R., Chao L., Xu G., Zhang Y., Zhou G., Yin D., Guo Y., Fu Y., Yang Y. (2024). RNAGenesis: A Generalist Foundation Model for Functional RNA Therapeutics. bioRxiv.

[47] Koblan L.W., Doman J.L., Wilson C., Levy J.M., Tay T., Newby G.A., Maianti J.P., Raguram A., Liu D.R. (2018). Improving cytidine and adenine base editors by expression optimization and ancestral reconstruction. Nat. Biotechnol..

[48] Pannu J., Bloomfield D., MacKnight R., Hanke M.S., Zhu A., Gomes G., Cicero A., Inglesby T.V. (2025). Dual-use capabilities of concern of biological AI models. PLoS Comput. Biol..

[49] Bloomfield D., Pannu J., Zhu A.W., Ng M.Y., Lewis A., Bendavid E., Asch S.M., Hernandez-Boussard T., Cicero A., Inglesby T. (2024). AI and biosecurity: The need for governance. Science.

[50] Bonomi L., Huang Y., Ohno-Machado L. (2020). Privacy challenges and research opportunities for genomic data sharing. Nat. Genet..

[51] Brauneck A., Schmalhorst L., Kazemi Majdabadi M.M., Bakhtiari M., Völker U., Baumbach J., Baumbach L., Buchholtz G. (2023). Federated Machine Learning, Privacy-Enhancing Technologies, and Data Protection Laws in Medical Research: Scoping Review. J. Med. Internet Res..

[52] Boscarino N., Cartwright R.A., Fox K., Tsosie K.S. (2022). Federated learning and Indigenous genomic data sovereignty. Nat. Mach. Intell..

[53] Norori N., Hu Q., Aellen F.M., Faraci F.D., Tzovara A. (2021). Addressing bias in big data and AI for health care: A call for open science. Patterns.

[54] Yun T., Li H., Chang P.-C., Lin M.F., Carroll A., McLean C.Y. (2020). Accurate, scalable cohort variant calls using DeepVariant and GLnexus. Bioinformatics.

[55] Wang M., Zhang Z., Bedi A.S., Velasquez A., Guerra S., Lin-Gibson S., Cong L., Qu Y., Chakraborty S., Blewett M. (2025). A call for built-in biosecurity safeguards for generative AI tools. Nat. Biotechnol..

[56] Zhang Z., Zhou Z., Jin R., Cong L., Wang M. (2025). GeneBreaker: Jailbreak Attacks against DNA Language Models with Pathogenicity Guidance. arXiv.

[57] Zhang Z., Chakraborty S., Bedi A.S., Mathew E., Saravanan V., Cong L., Velasquez A., Lin-Gibson S., Blewett M., Hendrycs D. (2025). Generative AI for Biosciences: Emerging Threats and Roadmap to Biosecurity. arXiv.

[58] Gisselbaek M., Berger-Estilita J., Devos A., Ingrassia P.L., Dieckmann P., Saxena S. (2025). Bridging the gap between scientists and clinicians: Addressing collaboration challenges in clinical AI integration. BMC Anesthesiol..

[59] Costa C., Silva J., Azevedo L.F., de Lemos M.S., Paneque M. (2024). A collaborative model for Medical Genetics services delivery in Portugal: A multidisciplinary perspective. J. Community Genet..

[60] Tang L., Li J., Fantus S. (2023). Medical artificial intelligence ethics: A systematic review of empirical studies. Digit. Health.

[61] Papachristou K., Katsakiori P.F., Papadimitroulas P., Strigari L., Kagadis G.C. (2024). Digital Twins’ Advancements and Applications in Healthcare, Towards Precision Medicine. J. Pers. Med..

[62] Venkatesh K.P., Raza M.M., Kvedar J.C. (2022). Health digital twins as tools for precision medicine: Considerations for computation, implementation, and regulation. NPJ Digit. Med..

[63] Lipkova J., Chen R.J., Chen B., Lu M.Y., Barbieri M., Shao D., Vaidya A.J., Chen C., Zhuang L., Williamson D.F.K. (2022). Artificial intelligence for multimodal data integration in oncology. Cancer Cell.

[64] Zhang Y., Zhang K., Prakosa A., James C., Zimmerman S.L., Carrick R., Sung E., Gasperetti A., Tichnell C., Murray B. (2023). Predicting Ventricular Tachycardia Circuits in Patients with Arrhythmogenic Right Ventricular Cardiomyopathy using Genotype-specific Heart Digital Twins. medRxiv.

[65] Simon B.D., Ozyoruk K.B., Gelikman D.G., Harmon S.A., Türkbey B. (2025). The future of multimodal artificial intelligence models for integrating imaging and clinical metadata: A narrative review. Diagn. Interv. Radiol..

[66] Zhuang H. (2025). How genomics and multi-modal AI are reshaping precision medicine. Front. Med..

[67] Lekadir K., Frangi A.F., Porras A.R., Glocker B., Cintas C., Langlotz C.P., Weicken E., Asselbergs F.W., Prior F., Collins G.S. (2025). FUTURE-AI: International consensus guideline for trustworthy and deployable artificial intelligence in healthcare. BMJ.

